# A High-Aperture-Efficiency Fabry–Perot Antenna with Broadband Out-of-Band RCS Reduction Enabled by a Partially Reflective Absorptive Frequency-Selective Surface

**DOI:** 10.3390/mi17091097

**Published:** 2026-09-18

**Authors:** Binbin Jiang, Yi-Feng Cheng, Dayong Gong, Yufeng Wang, Jiang Xiong, Yufeng Yu

**Affiliations:** 1 Zhejiang Key Laboratory of Quantum Materials and Control, School of Information and Electrical Engineering, Hangzhou City University, Hangzhou 310015, China; jiangbinbin97@gmail.com; 2 State Key Laboratory of Millimeter Waves, Southeast University, Nanjing 210096, China; chengyifeng2013@gmail.com; 3 School of Electronics and Information Engineering, Hangzhou Dianzi University, Hangzhou 310018, China; 4 Hangzhou ZoanRel Electronics Co., Ltd., Hangzhou 311308, China; g-gdy@outlook.com; 5 Jiaxing Novelidea Communication Technology Co., Ltd., Jiaxing 314001, China; wyf820925@hrbeu.cn; 6 Computational Electromagnetics Laboratory, Institute of Applied Physics, University of Electronic Science and Technology of China, Chengdu 611731, China; xiongjiang@uestc.edu.cn

**Keywords:** Fabry–Perot antenna, partially reflective absorptive frequency selective surface, radar cross section, aperture efficiency

## Abstract

A high-aperture-efficiency Fabry–Perot (FP) antenna with broadband out-of-band radar cross-section (RCS) reduction enabled by a partially reflective absorptive frequency-selective surface (PRAFSS) is proposed. Unlike conventional absorptive frequency-selective surfaces that emphasize high in-band transmission, the PRAFSS is designed specifically for FP cavity operation. It provides controlled reflection and transmission near 1 GHz to sustain cavity resonance, while providing broadband absorption at higher frequencies for out-of-band scattering suppression. A prototype is designed, fabricated, and measured. The measured −10 dB impedance bandwidth is 965–1009 MHz (4.46%), and the measured gain reaches approximately 13.0 dBi near 985 MHz, with a gain enhancement greater than 2.2 dB relative to the reference antenna. Using the measured gain and the exact 416 mm × 416 mm physical aperture, the measured gain-based aperture efficiency is approximately 85.1%. Full-wave far-field simulations predict a continuous 10 dB monostatic RCS reduction band of 3.05–6.47 GHz. The finite-range measurement shows a consistent reference-normalized backscattering reduction band of 3.15–6.32 GHz. The results demonstrate a cavity-oriented design strategy in which high aperture utilization in the antenna operating band and broadband out-of-band scattering suppression are implemented by different electromagnetic responses of the same PRAFSS.

## 1. Introduction

Fabry–Perot (FP) antennas have attracted considerable attention in wireless communication, radar, and sensing systems, owing to their advantages of simple configuration, low profile, and gain enhancement capability [1,2,3]. By introducing a partially reflective surface (PRS) above a primary radiator, multiple reflections are generated inside the cavity, resulting in enhanced directivity and gain. Compared with conventional antenna arrays, FP antennas can provide moderate gain enhancement without requiring complicated feeding networks, making them attractive for practical engineering applications [4,5].

Despite these advantages, conventional FP antennas usually exhibit relatively high radar cross-section (RCS) because of their large metallic ground plane and superstrate structures [6]. For modern electromagnetic systems, especially those operating in increasingly congested electromagnetic environments, reducing antenna scattering while maintaining satisfactory radiation performance has become an important design objective [7]. Consequently, low-RCS FP antennas have become an active research topic in recent years.

Low-scattering FP antennas have been implemented using phase-cancellation, coding, phase-gradient, and absorptive surfaces [8,9,10,11]. Related absorptive and rasorber concepts for antenna-scattering control include absorptive frequency-selective surfaces, frequency-selective rasorbers, and absorptive metasurfaces [12,13,14,15,16,17]. Representative Fabry–Perot and low-scattering FP developments span conventional cavity architectures and absorptive or phase-engineered superstrates [18,19,20,21,22,23,24,25,26,27,28]. Absorptive and partially reflective configurations are particularly relevant because they can suppress scattering without relying solely on angular redistribution. Representative examples include partially reflecting and absorbing surfaces [19,24,25], absorptive/transmissive frequency-selective structures [27], frequency-selective rasorber-based superstrates [22], and the recent absorptive partially reflective surface (APRS) reported in [28]. These studies establish that the general combination of absorption and partial reflection in an FP antenna is not, itself, new.

The present work therefore focuses on a narrower design objective: improving aperture utilization in a compact FP aperture while retaining a separate broadband out-of-band absorption function. In our previous ATFSS-based design [27], the antenna operating band was created as a transmission window of a bandpass FSS, and the FP effect relied on the residual reflection of that transmission-oriented structure. Such a passband-first approach is useful when high transmission is the primary requirement, but the transmission window circuitry is not essential for the single-polarized feed considered here and constrains the reflection magnitude and phase that can be used for cavity design.

In this work, the PRAFSS is instead designed from a cavity-oriented perspective. Around the antenna operating band, the reflection magnitude and phase are treated directly as FP cavity design variables, rather than by-products of a high-transmission window; only about 4.3% of the power incident from the antenna side is absorbed at 985 MHz. At higher frequencies, the same structure provides the absorption required for out-of-band scattering suppression. This frequency-separated functionality distinguishes the present design objective from conventional transmission-oriented AFSS/ATFSS radomes and from recent phase-cancellation FP antennas. Recent developments in chessboard frequency-selective rasorbers [29] and low-cost additive FP antennas [30] further illustrate the growing diversity of FP design objectives; the emphasis here is specifically compact-aperture utilization together with broadband out-of-band RCS reduction.

The fabricated antenna operates around 1 GHz, with a measured −10 dB impedance bandwidth of 965–1009 MHz (4.46%), a peak measured gain of approximately 13.0 dBi, and a measured gain enhancement greater than 2.2 dB relative to the reference antenna. The exact aperture is 1.367λ_0_ × 1.367λ_0_ at 985 MHz, giving a measured gain-based aperture efficiency of approximately 85.1%. The scattering function is intentionally out of band: full-wave far-field simulations give a continuous 10 dB monostatic RCS reduction band of 3.05–6.47 GHz, while finite-range measurements provide consistent comparative backscattering reduction behavior.

## 2. Antenna Configuration and Design Principle

### 2.1. Configuration of the Proposed FP Antenna

**Figure 1:**
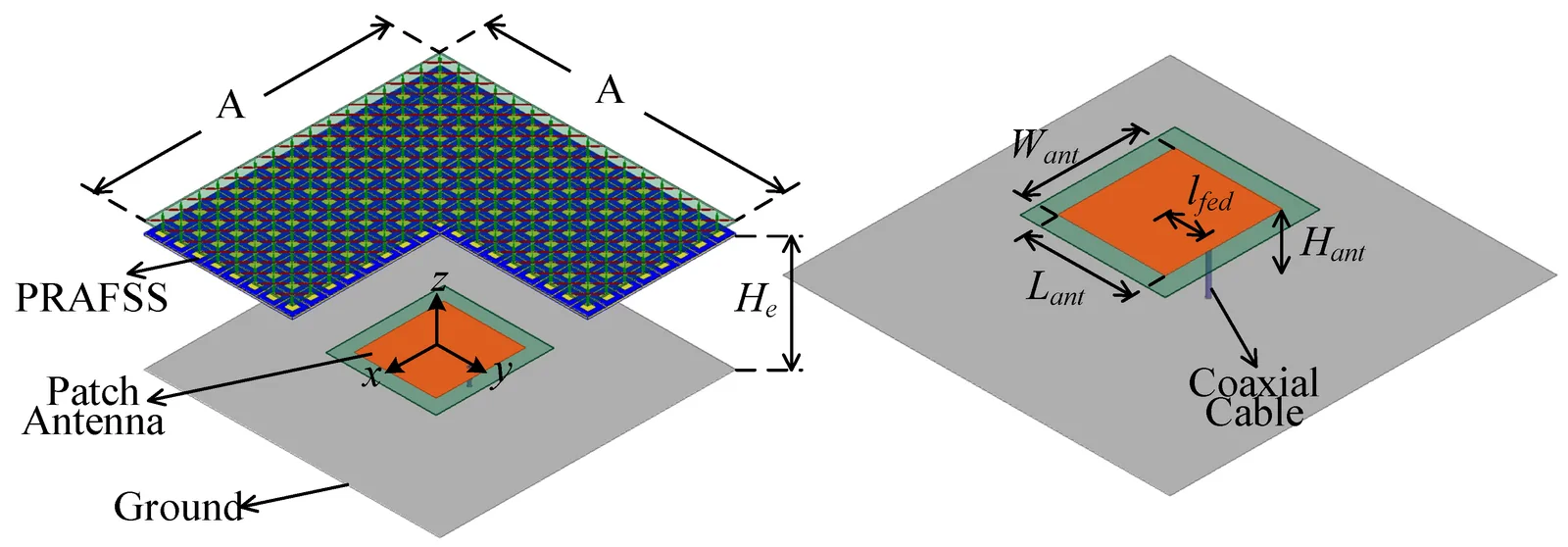
Geometry of the proposed FP antenna. *A* = 416 mm, *H_e_* = 165 mm, *W_ant_* = 126 mm, *H_ant_* = 12 mm, *L_ant_* = 125 mm, and *l_fed_* = 41 mm.

Figure 1 shows the geometry of the proposed FP antenna. The antenna comprises a PRAFSS, a coaxially fed microstrip patch antenna, and a 416 mm × 416 mm metallic ground plane. The PRAFSS and the large metallic ground plane form the global FP cavity, while the complete feed antenna is suspended inside the cavity.

The feed antenna is a conventional coaxially fed microstrip patch antenna with its own local ground plane and dielectric substrate. The patch is printed on a Rogers RT/duroid 5880 substrate (*ε_r_* = 2.2, tan*δ* = 0.0009) with a thickness of 1.016 mm; the substrate/local ground dimensions are 166 mm × 165 mm, and the copper thickness is 0.035 mm. The coaxial feed is located on the *y*-axis at *l_fed_* = 41 mm from the patch center. In the HFSS full-antenna model, the coaxial excitation is defined by a wave port. The feed antenna is located *H_ant_* = 12 mm above the large metallic ground plane, while *H_e_* denotes the distance from the lower surface of the partially reflective FSS to the large ground plane.

The reference antenna used throughout this work is obtained by removing the PRAFSS from the proposed FP antenna. Therefore, the reference antenna consists of the same patch antenna and the same large metallic ground plane. In this manner, both antennas possess the same physical aperture determined by the cavity ground plane, allowing the gain enhancement and RCS reduction introduced by the PRAFSS to be evaluated fairly without the influence of aperture size variation.

The PRAFSS serves simultaneously as the partially reflective surface required for FP cavity resonance and the absorptive structure responsible for scattering suppression. The detailed configuration and operating principle of the PRAFSS are presented in the following subsection.

### 2.2. Configuration and Design Principle of the PRAFSS

**Figure 2:**
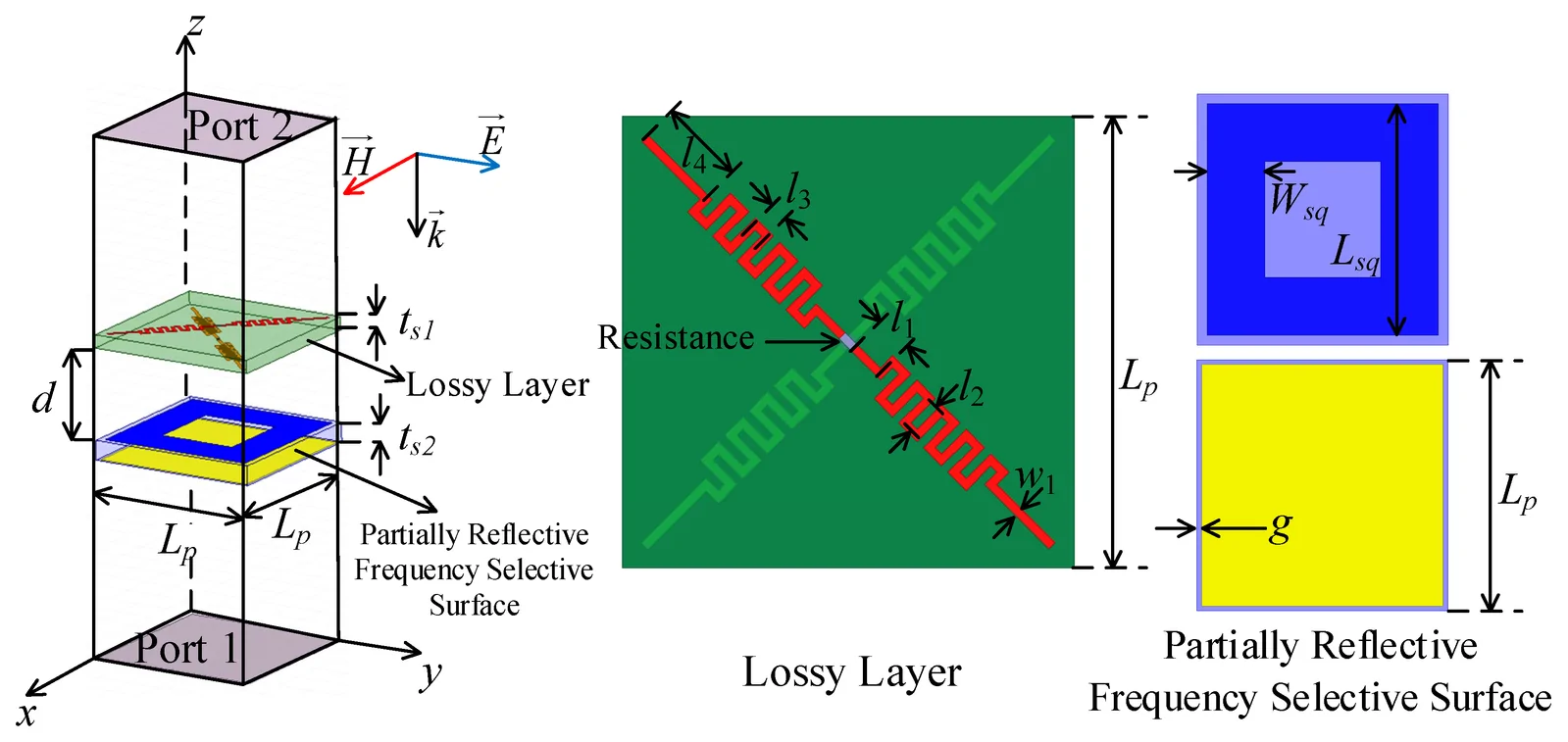
PRAFSS configuration. *L_p_* = 26 mm, *d* = 14 mm, *l*_1_ = 2.1 mm, *l*_2_ = 2 mm, *l*_3_ = 1 mm, *l*_4_ = 5 mm, *w*_1_ = 0.5 mm, *t_s_*_1_ = 2 mm, *L_sq_* = 24 mm, *W_sq_* = 6 mm, *g* = 0.5 mm, and *t_s_*_2_ = 3 mm.

Figure 2 depicts the unit cell configuration of the PRAFSS. It consists of a resistively loaded lossy layer and a partially reflective FSS separated by a 14 mm air gap (*d*) in the electromagnetic model. The lossy layer substrate is Rogers RT/duroid 5880 (*ε_r_* = 2.2, tan*δ* = 0.0009, *t_s_*_1_ = 2 mm), whereas the partially reflective FSS is printed on an F4B substrate (*ε_r_* = 2.65, tan*δ* = 0.0007, *t_s_*_2_ = 3 mm). The printed metallic patterns are modeled as 0.035 mm thick finite-conductivity copper. The periodicity of the unit cell is *L_p_* = 26 mm.

The proposed PRAFSS adopts a cavity-oriented design emphasis that differs from the transmission-oriented strategy commonly used for conventional absorptive frequency-selective surfaces (AFSSs). Traditional AFSS structures are primarily developed for radome and low-scattering applications, where high transmission within the operating band is generally preferred in order to minimize insertion loss. Such a transmission-oriented strategy is suitable for preserving antenna radiation performance, whereas the present FP design treats the in-band reflection magnitude and phase directly as cavity design variables.

Unlike conventional antennas, gain enhancement in FP antennas relies on cavity resonance established between the metallic ground plane and the superstrate. Consequently, the superstrate must provide an appropriate reflection coefficient to sustain resonance. Excessive transmission weakens the cavity effect, whereas excessive absorption introduces radiation loss. Therefore, an inherent tradeoff exists among gain enhancement, aperture efficiency, and RCS reduction.

To address this challenge, the proposed PRAFSS is designed according to a cavity-oriented philosophy. Instead of maximizing transmission within the operating band, the PRAFSS intentionally provides controlled reflection and transmission characteristics. The reflected wave contributes to the establishment of cavity resonance, whereas the transmitted wave forms the desired radiation. At higher frequencies, strong absorption is introduced to suppress electromagnetic scattering. In this manner, the PRAFSS simultaneously fulfills the dual functions of gain enhancement and RCS reduction.

### 2.3. Electromagnetic Characteristics of the PRAFSS

All full-wave simulations were performed using Ansys HFSS 2022 R1. For the PRAFSS unit cell, periodic boundaries are applied along the *x*- and *y*-directions, and Floquet ports are assigned along *z*. Port 1 is on the antenna-facing side adjacent to the partially reflective FSS, and Port 2 is on the external illumination side adjacent to the lossy layer. The ports are de-embedded to the lower surface of the partially reflective FSS and the upper surface of the lossy layer, respectively. Thus, *S*_11_ and *S*_21_ characterize incidence from the antenna side, whereas *S*_22_ and *S*_12_ characterize external illumination from the lossy layer side. Under normal incidence, the two orthogonal fundamental excitations are denoted *x*- and *y*-polarized waves; TE/TM notation is used only for oblique incidence after the plane of incidence is defined. Cross-polarized fundamental S-parameters are negligible, and no higher-order propagating Floquet modes exist over the frequency range used for the normal-incidence absorption analysis.

**Figure 3:**
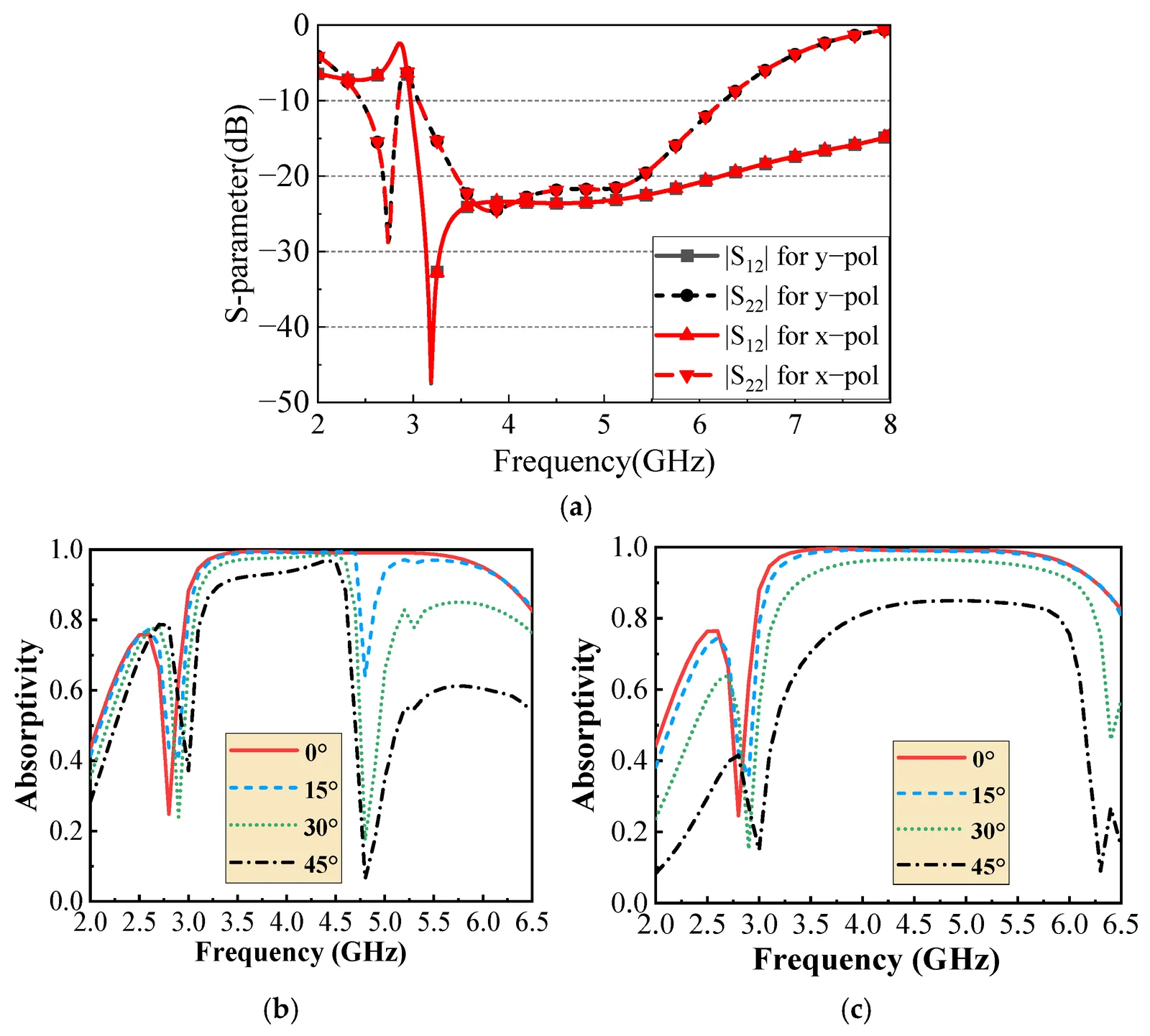
Electromagnetic response of the PRAFSS for external illumination from Port 2: (**a**) normal-incidence reflection/transmission characteristics; (**b**) TE-polarized absorptivity at *θ* = 0°, 15°, 30°, and 45° over 2–6.5 GHz; (**c**) TM-polarized absorptivity at *θ* = 0°, 15°, 30°, and 45° over 2–6.5 GHz.

Figure 3 shows the electromagnetic response for external illumination from Port 2. Under normal incidence, the *x*- and *y*-polarized responses are essentially identical. Because the cross-polarized fundamental terms are negligible, and no higher-order propagating Floquet modes are present in the considered normal-incidence band, strong broadband absorption is obtained in the 3–6 GHz region. For the oblique-incidence analysis, the *x*–*z* plane (φ = 0°) is selected as the incidence plane; owing to the symmetry of the unit cell with respect to the two principal axes, the corresponding *y*–*z*-plane response is equivalent. Additional TE- and TM-polarized simulations were performed for incidence angles of 0°, 15°, 30°, and 45°. The oblique-incidence results in Figure 3b,c are limited to 2–6.5 GHz, below the onset of higher-order propagating Floquet modes for all investigated incidence angles up to 45°. Within this range, only the fundamental modes propagate, and the cross-polarized fundamental components remain negligible. The absorptivity is therefore evaluated from the co-polarized fundamental reflected and transmitted powers as follows:

$$A_{ext}=1-{\left|S_{22}\right|}^{2}-{\left|S_{12}\right|}^{2}$$

The TM response remains comparatively stable up to 30°, whereas the TE response is more angle-sensitive. In particular, at 4.8 GHz, the TE absorptivity decreases from 0.991 at normal incidence to 0.639, 0.174, and 0.064 at 15°, 30°, and 45°, respectively. The transmission remains low near this frequency while the reflection of the complete PRAFSS increases. Additional simulations of the partially reflective FSS alone show a pronounced TE angular variation around 4.8 GHz, indicating that the local absorption degradation is mainly associated with the angle-dependent FSS response perturbing the impedance-matching condition of the composite PRAFSS. Therefore, polarization-insensitive broadband absorption is claimed primarily for normal incidence, and the limited TE angular stability is explicitly acknowledged.

**Figure 4:**
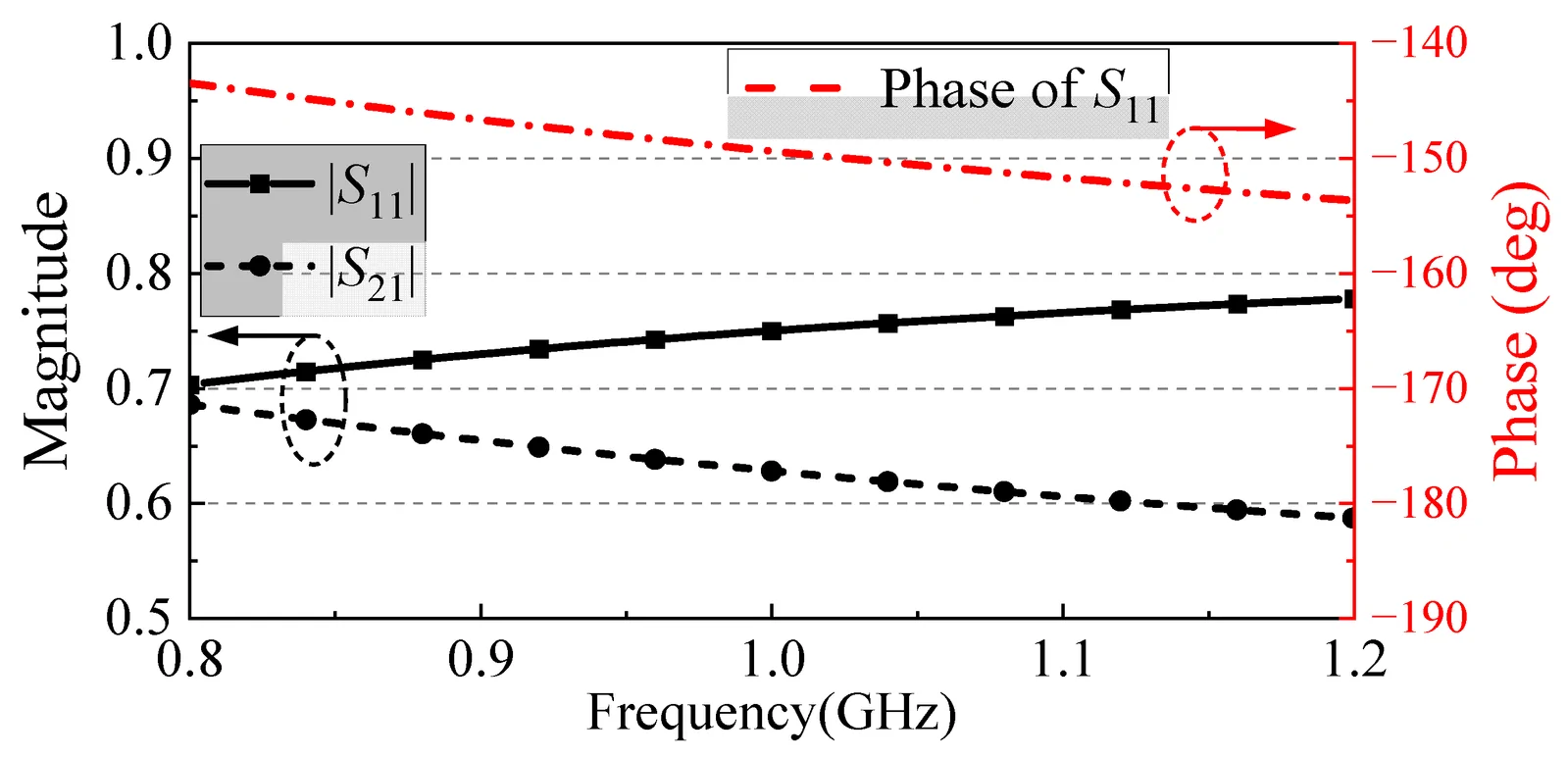
Simulated reflection magnitude |*S*_11_|, transmission magnitude |*S*_21_|, and reflection phase ∠*S*_11_ of the PRAFSS for incidence from Port 1 around the antenna operating frequency.

The antenna-side response around the operating frequency is shown in Figure 4. Here, *S*_11_ and *S*_21_ are the reflection and transmission coefficients for incidence from Port 1, and the *S*_11_ phase is referenced to the lower surface of the partially reflective FSS through port de-embedding. At 985 MHz, |*S*_11_| = 0.747, |*S*_21_| = 0.632, and ∠*S*_11_ ≈ −149°. The corresponding reflected and transmitted power fractions are 0.558 and 0.399, respectively, giving an absorbed fraction *A_ant_* = 1 − |*S*_11_|^2^ − |*S*_21_|^2^ ≈ 0.043. Thus, only approximately 4.3% of the power incident on the PRAFSS from the antenna side is dissipated in the PRAFSS at the center frequency, while substantial reflection is retained to sustain the FP resonance.

These results confirm that the PRAFSS behaves as a low-loss partially reflective surface in the antenna operating band, rather than as a high-transmission window. The reflected component provides cavity feedback, the transmitted component contributes to forward radiation, and only a small fraction is dissipated by the lossy layer.

Figure 3 and Figure 4 therefore show two frequency-separated functions of the same PRAFSS: controlled partial reflection with low absorption around the 1 GHz antenna band, and strong absorption in the higher-frequency region used for out-of-band scattering suppression.

For the complete antenna, radiation boundaries are used. HFSS adaptive meshing is employed with a maximum ΔS convergence criterion of 0.02 and a maximum of eight adaptive passes. Copper is modeled with finite conductivity, the 300 Ω chip resistors are represented by lumped resistive elements, and all antenna/PRAFSS layers are concentrically aligned in the full model.

## 3. Simulated Results and Discussion

### 3.1. Reflection and Radiation Characteristics

**Figure 5:**
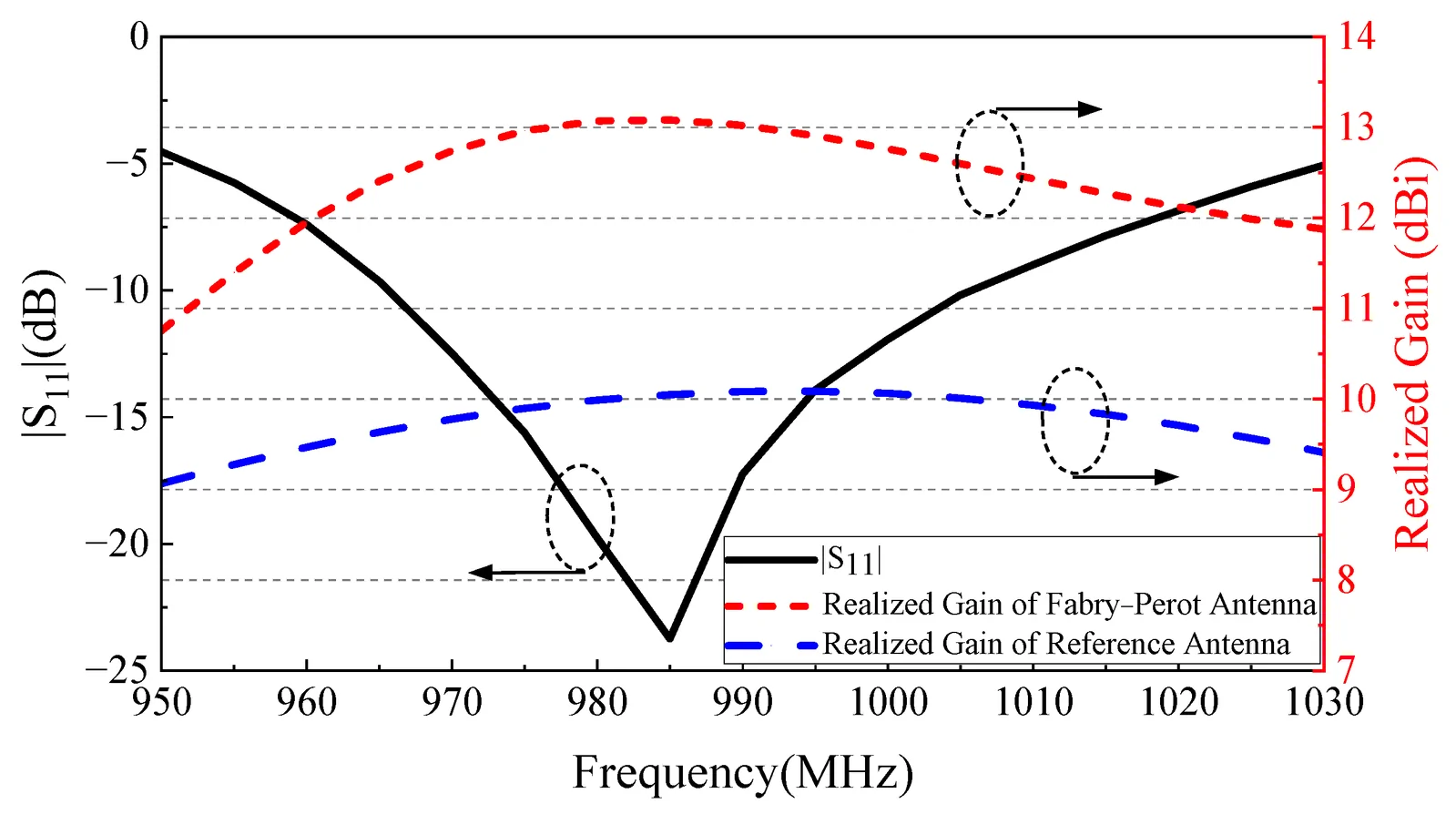
Simulated |S11| and realized gain of the proposed FP antenna and the reference antenna.

The reflection and radiation characteristics of the proposed FP antenna are investigated using the complete full-wave model. Figure 5 compares the proposed and reference antennas. The simulated −10 dB impedance bandwidth of the proposed antenna is 965–1005 MHz, corresponding to 4.1%.

Figure 5 also compares the simulated realized gains. The proposed FP antenna exhibits a gain enhancement greater than 2.3 dB over the simulated operating band, with a peak near the design frequency. The reference antenna uses the same feed antenna and the same large ground plane; therefore, the improvement is attributable to the FP resonance introduced by the PRAFSS, rather than to a change in physical aperture.

At 985 MHz, the simulated peak directivity, gain, and realized gain are 13.54 dBi, 13.17 dBi, and 13.11 dBi, respectively. The simulated radiation efficiency and total efficiency are 91.78% and 90.5%, respectively. In this work, the experimental aperture efficiency is evaluated using the measured gain. As clarified in Section 4, no additional impedance mismatch correction is applied in the gain-transfer measurement; therefore, the measured gain includes the terminal mismatch effect and corresponds physically to the realized gain quantity used in the HFSS simulations. The linear gain *G* is obtained from the gain in decibels as follows:

(1) $$G\;=\;10^{\frac{G_{dB}}{10}}$$

and the corresponding aperture efficiency is calculated as follows:

(2) $$\eta_{ap}\;=\;\frac{G{\lambda_{0}}^{2}}{4\pi A}\;\times \;100\%$$

where λ_0_ is the free-space wavelength and *A* is the physical aperture area. Using the measured gain of approximately 13.0 dBi and the exact 416 mm × 416 mm aperture yields *η_ap_* ≈ 85.1%. For reference, using the simulated directivity in Equation (2) gives a directivity-based aperture efficiency of approximately 96.2%. The latter excludes mismatch and dissipative losses and is provided only as a reference value.

**Figure 6:**
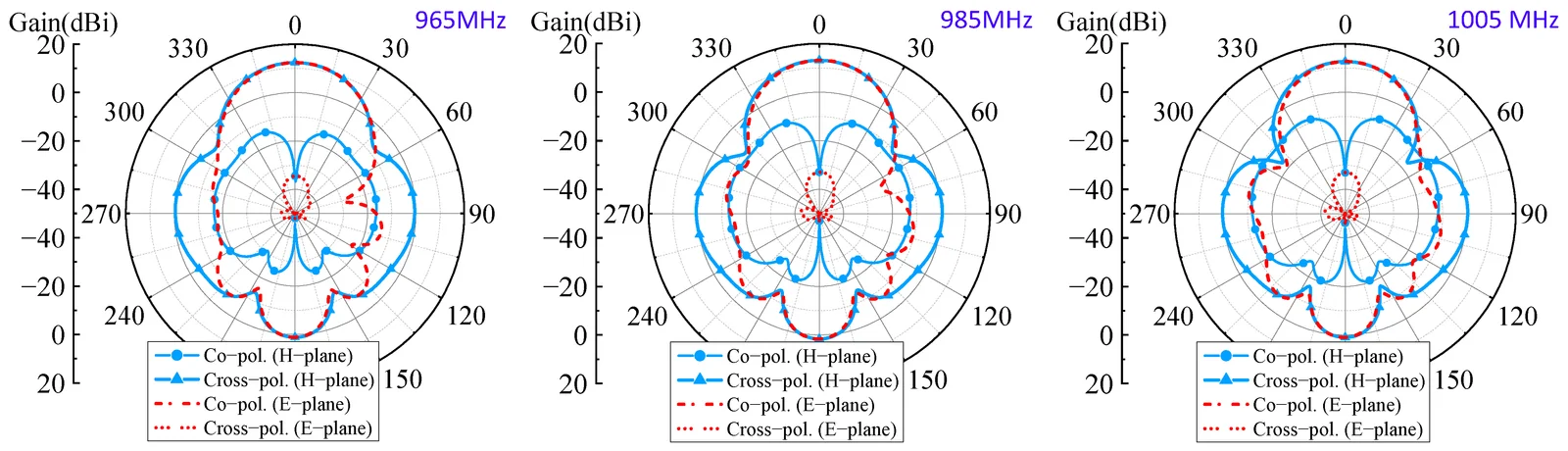
Simulated co− and cross−polarized radiation patterns of the proposed FP antenna at 965, 985, and 1005 MHz.

Figure 6 presents the simulated co- and cross-polarized radiation patterns at 965, 985, and 1005 MHz. Because the antenna is narrowband, the co-polarized pattern shape varies only slightly across the operating band. The simulated cross-polarization is very low in the main-beam region: in the *x*–*z* plane, it is more than 40 dB below the co-polarized main beam, and in the *y*–*z* plane, the broadside co-to-cross discrimination is also greater than 40 dB, although the discrimination decreases at large observation angles.

### 3.2. Out-of-Band RCS Reduction Performance

**Figure 7:**
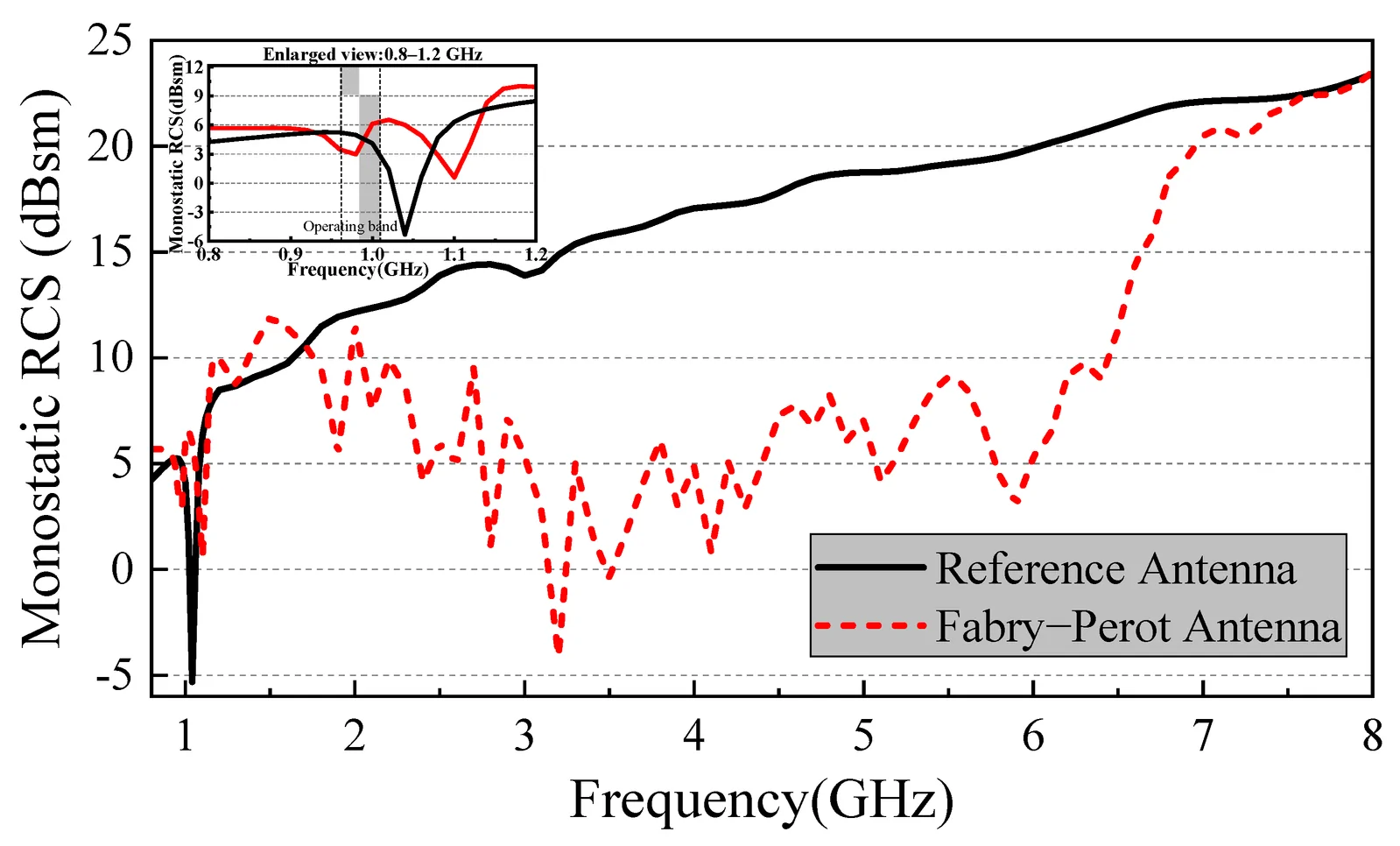
Simulated monostatic RCS of the proposed FP antenna and the reference antenna from 0.8 to 8 GHz under normal incidence.

Figure 7 compares the simulated monostatic RCS responses of the proposed and reference antennas over the extended frequency range of 0.8–8 GHz. Around the antenna operating band near 1 GHz, no sustained RCS reduction is observed. Below approximately 3 GHz, the RCS of the proposed antenna generally approaches that of the reference antenna, and a local RCS increase occurs around 1.5 GHz. This behavior is consistent with the intended frequency-selective operation of the PRAFSS: around 985 MHz, it is designed to provide controlled partial reflection with only about 4.3% absorption, rather than to act as an absorber. In-band RCS suppression is therefore not a design objective of the present antenna. In contrast, pronounced scattering suppression appears when the PRAFSS enters its higher-frequency absorption region. The simulated continuous 10 dB monostatic RCS reduction band is 3.05–6.47 GHz, corresponding to a fractional bandwidth of approximately 71.8%.

The 3.05–6.47 GHz reduction band agrees with the absorption region of the PRAFSS, supporting an absorption-dominated out-of-band suppression mechanism. Above approximately 6.5 GHz, the reduction gradually decreases as the absorptive response weakens. Accordingly, the RCS reduction performance is primarily confined to the out-of-band region, rather than the antenna operating band.

**Figure 8:**
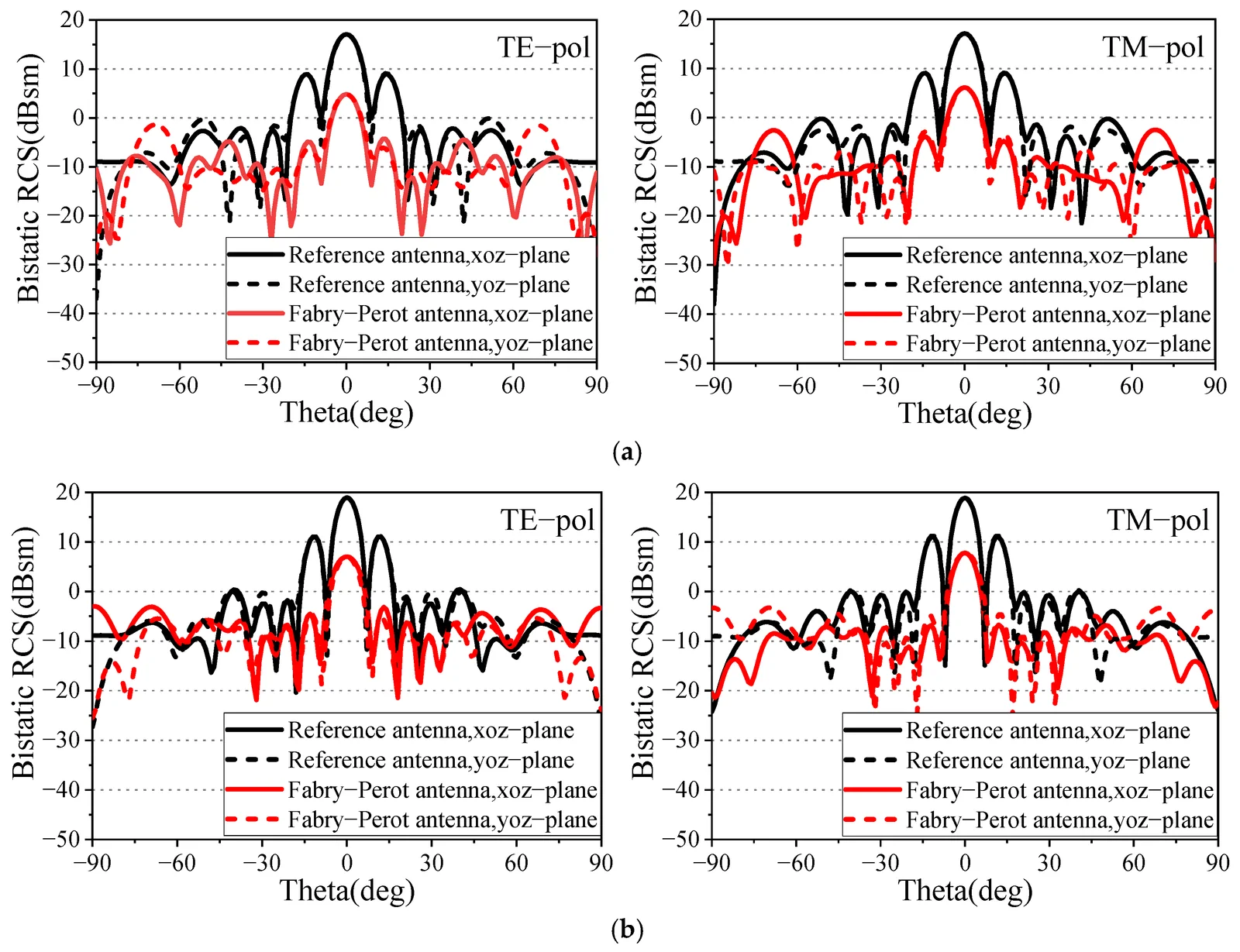
Simulated bistatic RCS of the proposed and reference antennas at (**a**) 4 GHz and (**b**) 5 GHz.

To further evaluate the scattering characteristics, bistatic RCS simulations were performed at 4 and 5 GHz. Figure 8 shows the bistatic RCS distributions in the xoz- and yoz-planes under TE- and TM-polarized incidences. Compared with the reference antenna, lower scattering levels are observed over most angles in the two investigated principal-plane cuts. The reduced scattering observed in the two investigated principal-plane cuts is consistent with the absorption-dominated mechanism of the PRAFSS.

### 3.3. Parametric Analysis and Influence of the Lossy Layer

The large 416 mm × 416 mm metallic ground plane is defined as the global lower reflecting boundary of the FP cavity. The smaller 166 mm × 165 mm ground plane belongs to the suspended feed antenna and occupies only the central region; it locally perturbs the cavity field but is not used as the lower reference plane in the first-order resonance calculation. The upper reference plane is the lower surface of the partially reflective FSS, which is also the de-embedded phase-reference plane used for *S*_11_ in Figure 4. The round-trip phase condition can therefore be written as *φ_PRAFSS_* + *φ_GND_* − 2*k*_0_*H_e_* = −2*N*π, where *k*_0_ = 2π/*λ*_0_. At 985 MHz, *λ*_0_ ≈ 304.4 mm, *φ_PRAFSS_* ≈ −149°, and the large metallic ground is approximated as a PEC reflector with *φ_GND_* = 180°. For *N* = 1, *H_e_* = [(−149° + 180°)/720° + 1/2]*λ*_0_ ≈ 165.3 mm, in close agreement with the adopted *H_e_* = 165 mm.

**Figure 9:**
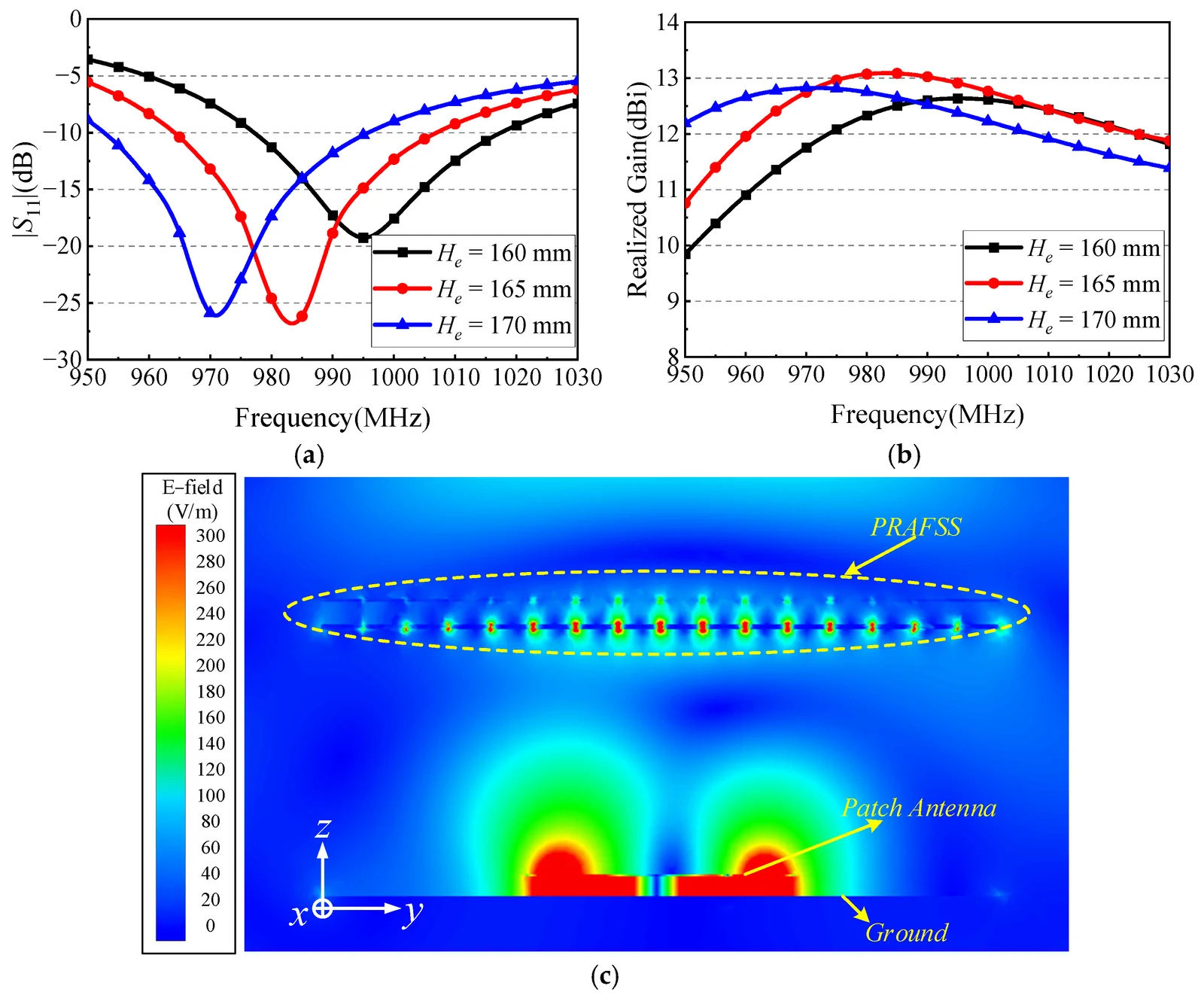
Simulated (**a**) reflection coefficients and (**b**) realized gains for different cavity heights He, and (**c**) electric field magnitude distribution in the central vertical plane at 985 MHz for *He* = 165 mm.

The analytical phase condition provides a first-order estimate for the dominant cavity formed over the full aperture. The practical structure is a compound cavity because the local feed ground perturbs the central field distribution; this effect is included explicitly in the complete full-wave model. Figure 9c shows the electric field magnitude distribution at 985 MHz for the final geometry. Together with the full-wave height sweep in Figure 9a,b, the field distribution confirms that the selected *H_e_* = 165 mm supports the intended resonant cavity behavior despite the localized feed–ground perturbation.

Among the three investigated cases, He = 165 mm provides the highest realized gain at 985 MHz. The simulated realized gains at this frequency are approximately 12.5, 13.0, and 12.6 dBi for He = 160, 165, and 170 mm, respectively, consistent with the quantitative phase calculation above.

**Figure 10:**
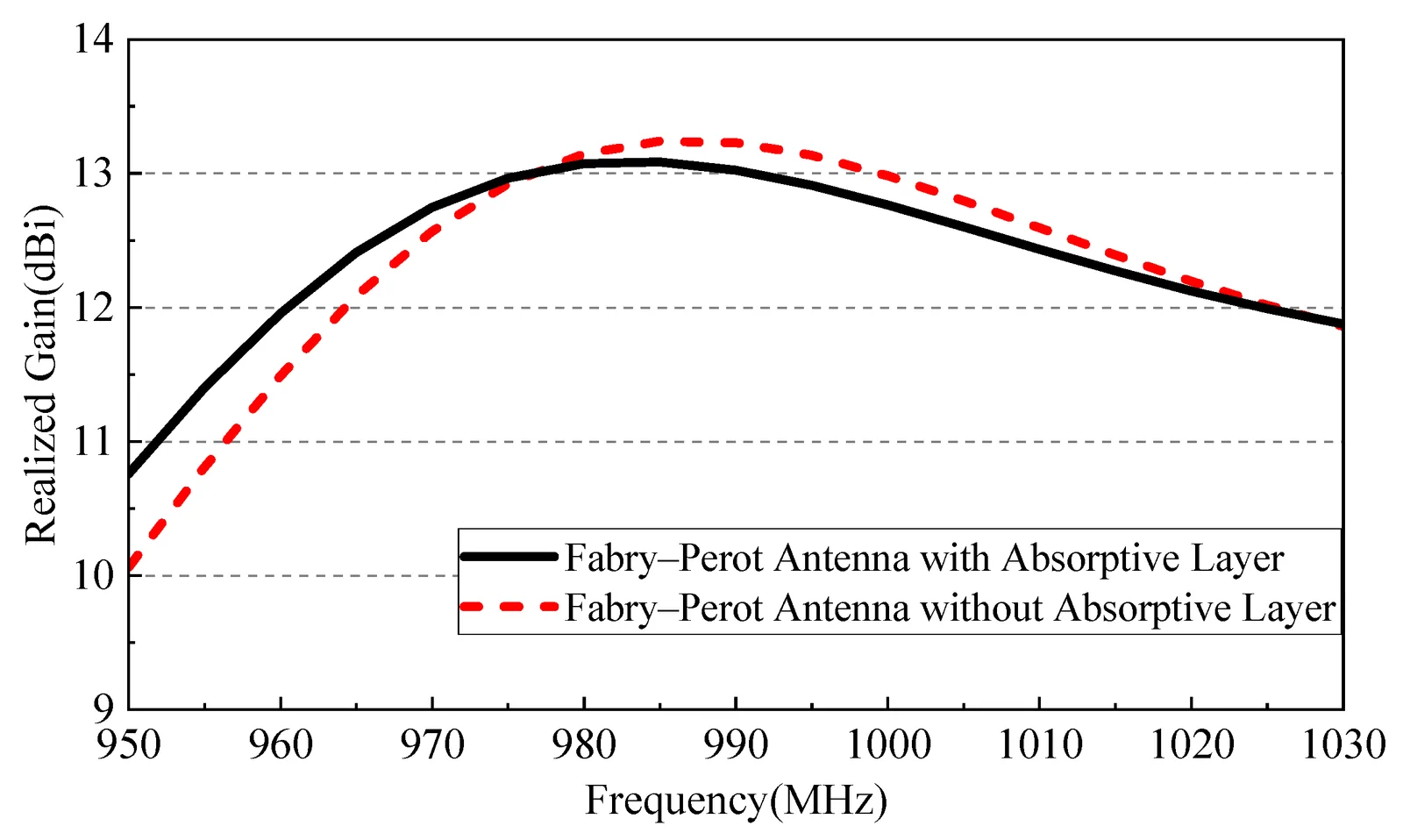
Simulated realized gains of the FP antenna with and without the absorptive layer.

Figure 10 compares the complete antenna with and without the entire lossy layer. This comparison quantifies the net influence of incorporating the lossy layer structure on antenna realized gain; it should not be interpreted as an isolated measurement of resistor dissipation alone because removing the full layer also changes the electromagnetic loading. The realized gain difference remains below 0.2 dB across the operating band, consistent with the 4.3% antenna-side absorption of the complete PRAFSS at 985 MHz and the high simulated radiation/total efficiencies reported above.

### 3.4. Discussion on Aperture Efficiency

**Figure 11:**
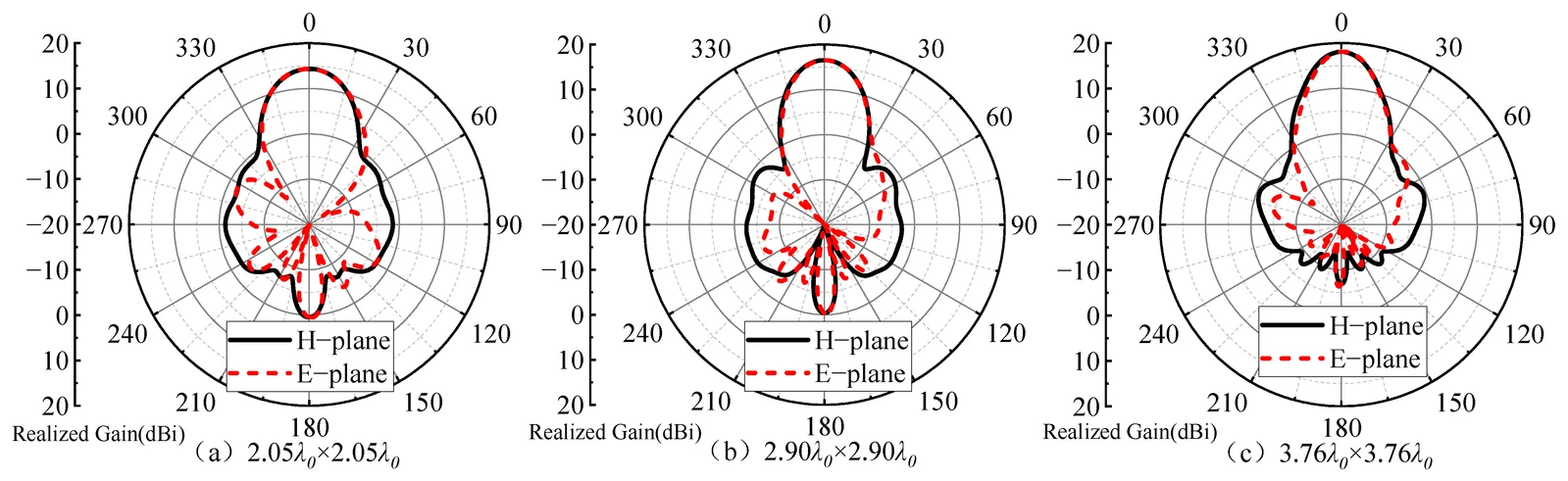
Simulated radiation patterns of FP antennas with different aperture sizes. (**a**) 2.05λ_0_ × 2.05λ_0_, (**b**) 2.9λ_0_ × 2.9λ_0_, (**c**) 3.76λ_0_ × 3.76λ_0_.

**Figure 12:**
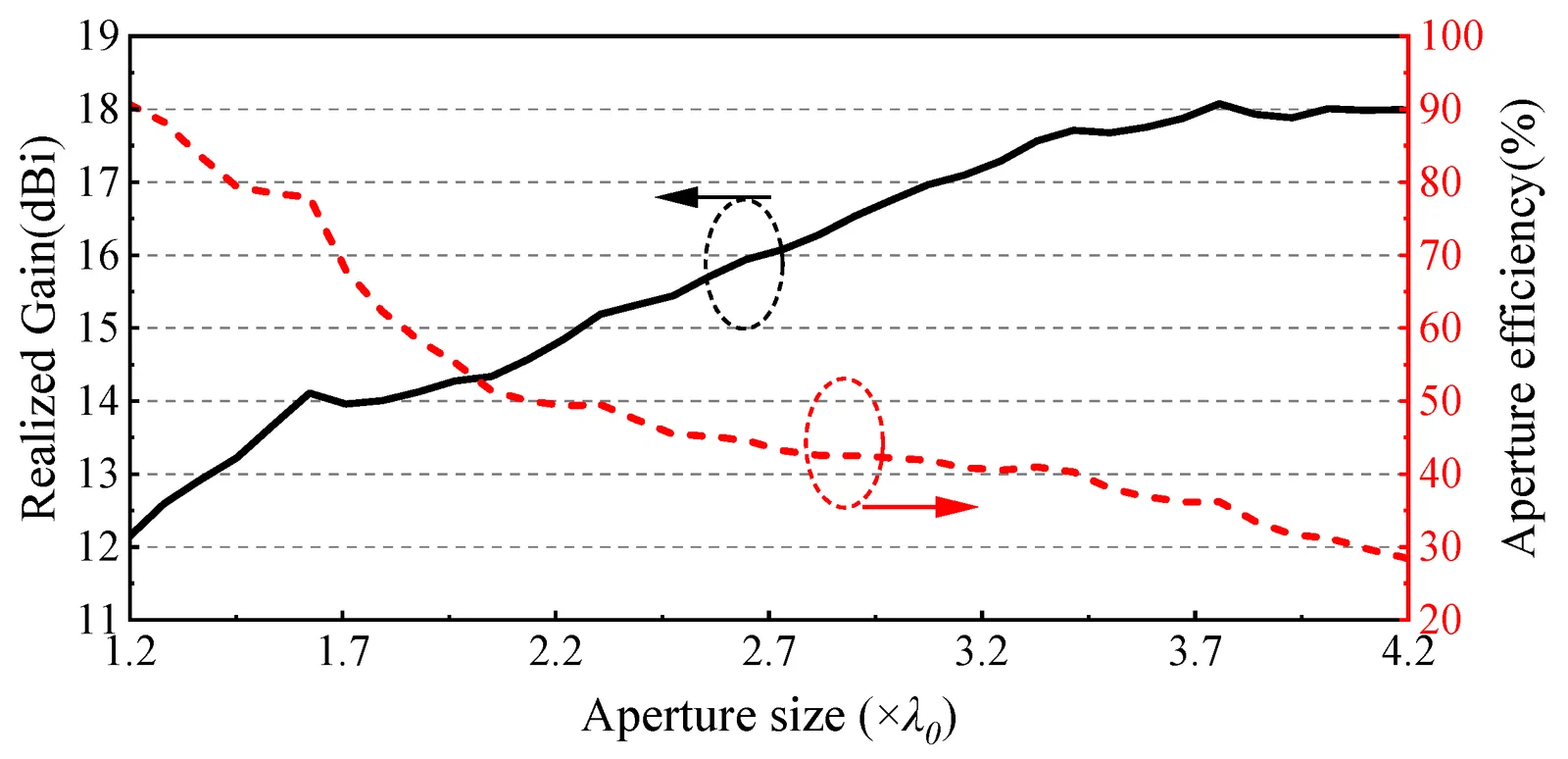
Simulated realized gain and aperture efficiency versus aperture size.

The realized gain and aperture efficiency of an FP antenna depend strongly on the electrical aperture size. Figure 11 compares antennas with progressively enlarged apertures at 985 MHz. As the aperture increases, the realized gain rises and the beamwidth narrows, whereas the aperture efficiency decreases. Figure 12 shows that the realized gain eventually tends to saturate beyond approximately 3.8λ_0_, while the aperture efficiency continues to fall. Thus, the high aperture efficiency of the final antenna should not be viewed as an aperture-independent material property; it reflects effective utilization of a comparatively compact aperture. Accordingly, the electrical aperture and total height should be considered together with aperture efficiency when comparing different FP antenna designs.

**Figure 13:**
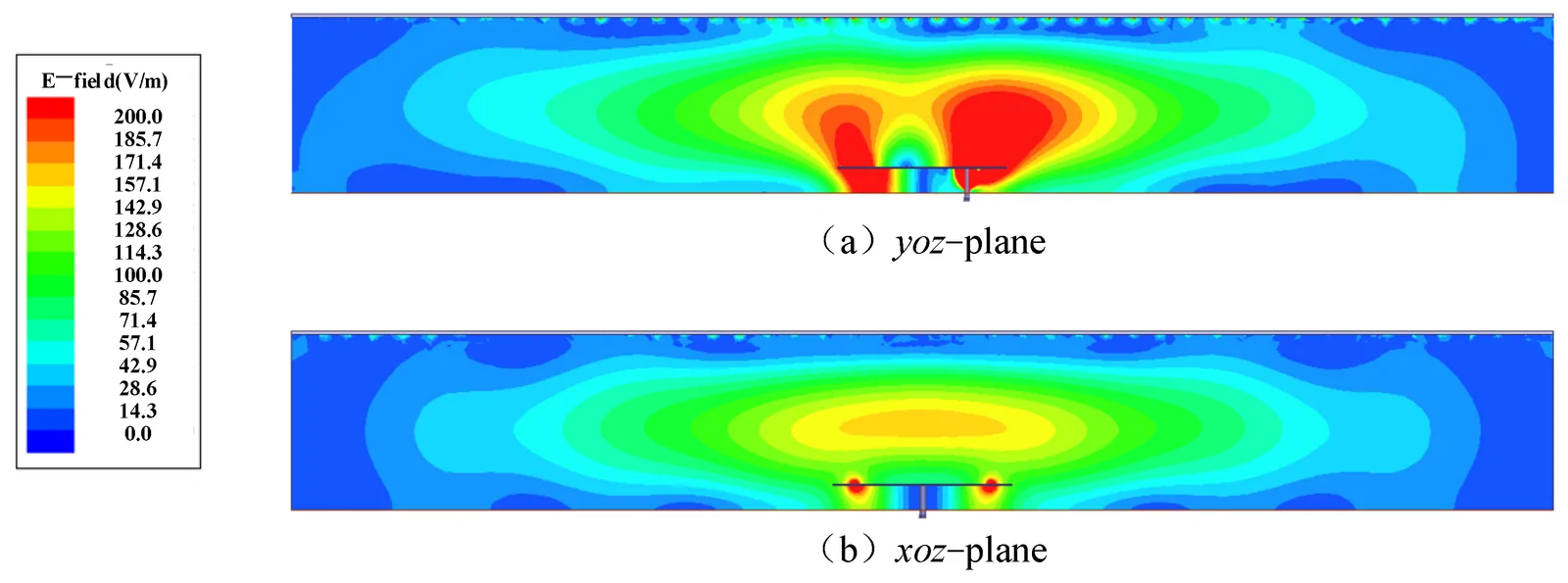
Electric field distribution at 985 MHz (3.76λ_0_ × 3.76λ_0_).

This size dependence is related to the nonuniform field distribution inside the FP cavity. Figure 13 shows the electric field distribution at 985 MHz for the largest investigated aperture. The field is concentrated mainly in the central region and decreases toward the edges; consequently, newly added outer aperture contributes progressively less to radiation, explaining why aperture efficiency decreases as the physical aperture is enlarged.

## 4. Experimental Validation

**Figure 14:**
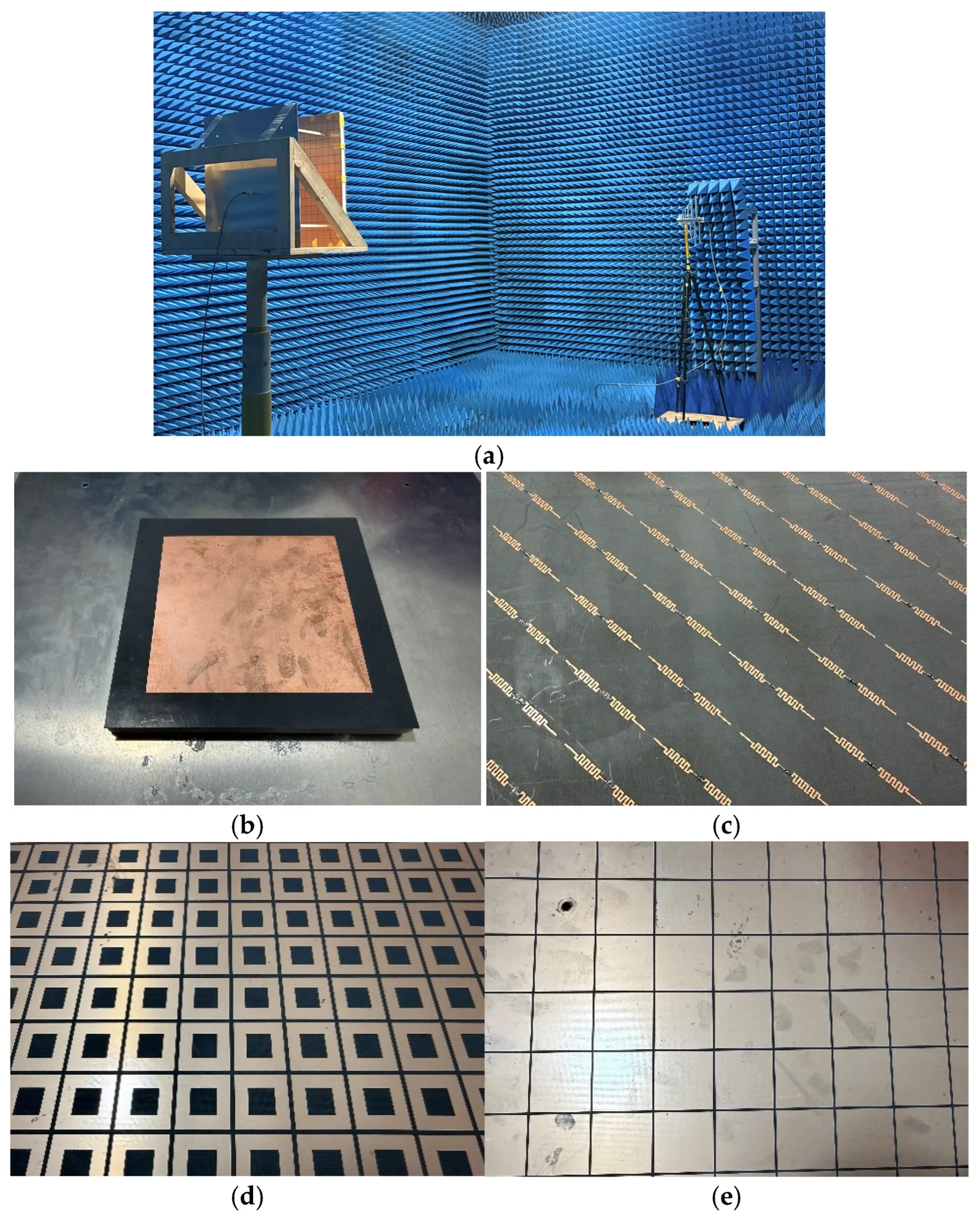
Prototype of the proposed FP antenna. (**a**) Proposed antenna under measurement; (**b**) antenna without PRAFSS; (**c**) lossy layer; (**d**) top view of the partially reflective FSS; and (**e**) bottom view of the partially reflective FSS.

A prototype of the proposed FP antenna was fabricated and experimentally characterized. The component photographs are shown in Figure 14. The fabricated PRAFSS contains 16 × 16 unit cells. Each lossy layer unit cell contains two 300 Ω chip resistors, with one resistor soldered to the metallic trace on each side of the Rogers 5880 substrate; therefore, a total of 512 resistors are used. In the electromagnetic model, the 14 mm interlayer region is air, whereas in the prototype, a 14 mm thick low-permittivity PMI foam (εr ≈ 1.05–1.10, tan δ = 0.005) is used to maintain the designed separation. The PRAFSS layers are primarily fixed by nylon bolts and nuts and are locally reinforced using Kafuter K-705 adhesive. The feed-antenna substrate, PMI spacer, and large metallic ground are also bonded using Kafuter K-705 adhesive. The adhesive is used as a thin local mechanical bonding layer and is not explicitly included in the electromagnetic model. Four 165 mm nylon supports maintain the cavity height. The total antenna dimensions are 416 mm × 416 mm × 186 mm, corresponding to an exact aperture of approximately 1.367λ_0_ × 1.367λ_0_ and a total height of approximately 0.61λ_0_ at 985 MHz.

The robustness of the PRAFSS to practical resistor variation was additionally evaluated. Varying the nominal 300 Ω resistance to 285 and 315 Ω (±5%) produces only negligible changes in the reflection/transmission response over the absorption band for both polarizations. A representative 0.7 nH series inductance was also added to each lumped resistor to model moderate soldering parasitics; the absorption-band response remains nearly unchanged. These simulations indicate that the broadband absorption is relatively insensitive to typical resistor tolerance and moderate series-inductance parasitics.

The antenna reflection coefficient, gain, and radiation patterns were measured using a Keysight E5063A ENA vector network analyzer (Keysight, Richardson, TX, USA) in an anechoic chamber. Gain was measured by the gain-transfer method at a Tx–AUT separation of 3 m using a calibrated broadband log-periodic dipole antenna as the reference. The calibrated reference value near 985 MHz is 6.32 dBi, with a calibration uncertainty of approximately 5%. Under identical measurement geometry and instrument settings, the AUT gain was obtained from the following:

$$G_{AUT,dB}=G_{std,dB}+20{log}_{10}\left|\frac{S_{21,AUT}}{S_{21,std}}\right|$$

**Figure 15:**
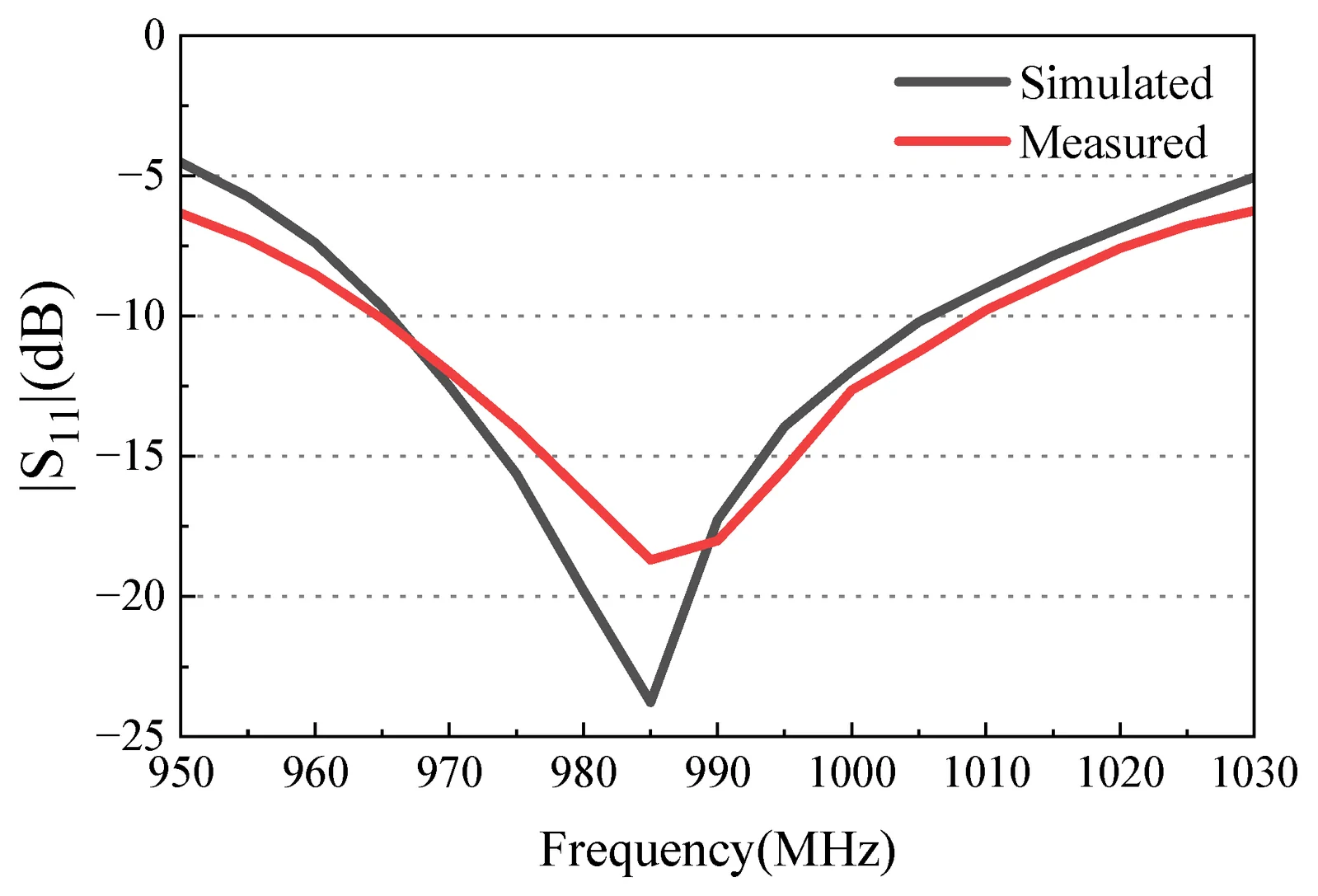
Simulated and measured |S_11_| of the FP antenna.

The calibrated reference value was used directly, and no additional impedance mismatch correction was applied to either the reference antenna or the AUT. Accordingly, the experimentally obtained quantity, referred to here as measured gain following common antenna measurement practice, includes the terminal mismatch effect and corresponds physically to the realized gain quantity used in the HFSS simulations. The estimated overall uncertainty of the gain-transfer measurement is approximately ±0.3 dB. Since the aperture efficiency is proportional to the linear gain, the nominal measured gain-based aperture efficiency of 85.1% corresponds to approximately 79.4–91.2% over this uncertainty range. As seen in Figure 15, the measured −10 dB impedance bandwidth is 965–1009 MHz (44 MHz, 4.46%), compared with the simulated 965–1005 MHz band (4.1%).

**Figure 16:**
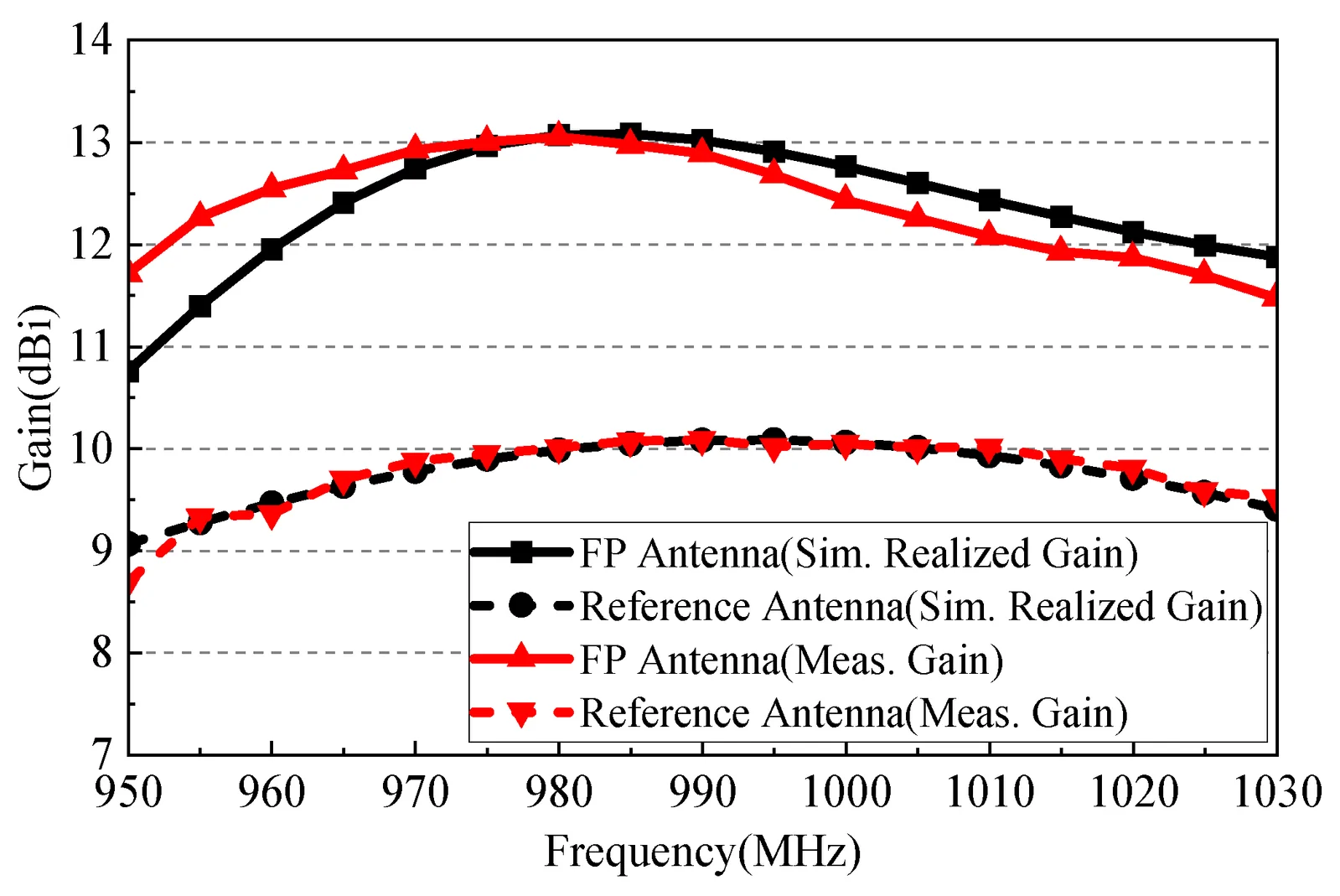
Simulated realized gain and measured gain of the proposed FP antenna and the reference antenna.

Figure 16 compares the measured gain and simulated realized gain of the proposed and reference antennas. The proposed antenna reaches a measured gain of approximately 13.0 dBi near 985 MHz and provides a gain enhancement greater than 2.2 dB relative to the reference antenna over the measured operating band. The measured gain and simulated–realized gain trends agree well.

**Figure 17:**
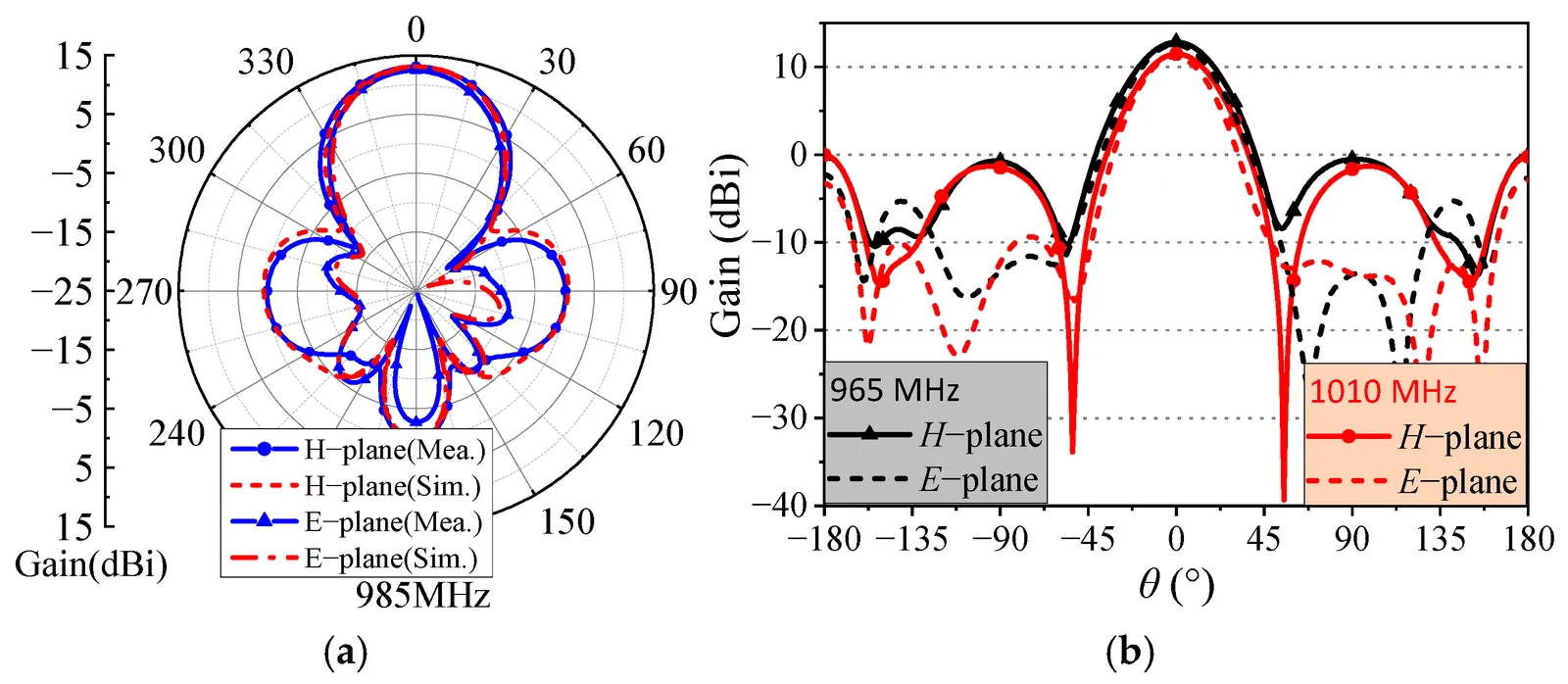
(**a**) Simulated and measured co-polarized radiation patterns at 985 MHz, and (**b**) measured co-polarized radiation patterns at 965 MHz and 1010 MHz.

Figure 17a shows the simulated and measured co-polarized patterns at 985 MHz, and Figure 17b presents measured patterns at 965 MHz and 1010 MHz. Because the radiation patterns were measured at 5 MHz intervals, 1010 MHz is the closest available frequency to the measured upper impedance-band edge of 1009 MHz. The measured patterns remain stable across this narrow operating band and are consistent with the simulated pattern stability shown in Figure 6. Only co-polarized patterns were recorded experimentally; the corresponding simulated cross-polarized results are provided in Figure 6.

**Figure 18:**
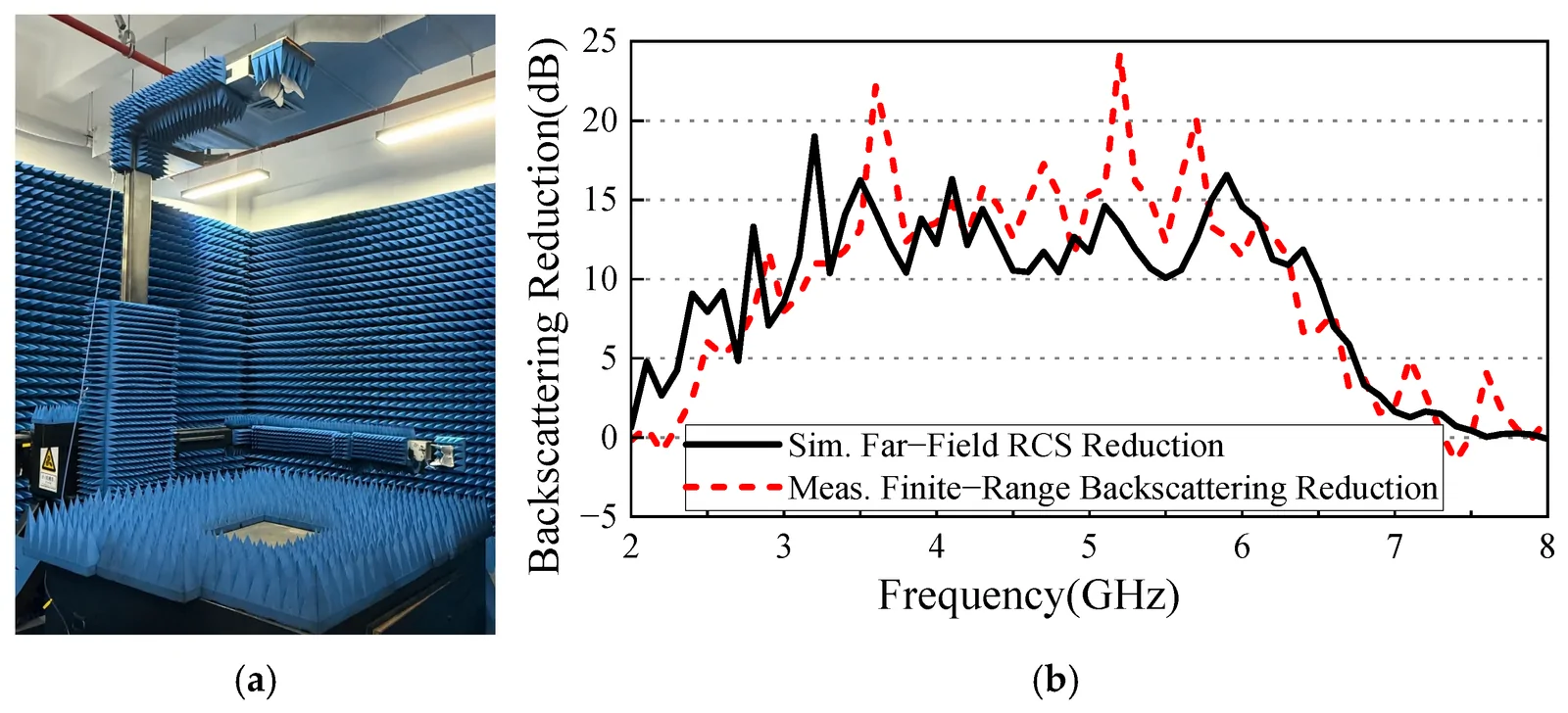
(**a**) Finite-range monostatic scattering measurement environment; (**b**) simulated far-field RCS reduction and measured finite-range backscattering reduction in the proposed FP antenna relative to the reference antenna.

The monostatic scattering measurement was performed using a one-port free-space reflection configuration with a broadband double-ridged horn connected to a Ceyear 3674G vector network analyzer (Ceyear Technologies, Qingdao, China), as shown in Figure 18a. The horn was used for both transmission and reception. The antenna under test was placed 2.5 m from the horn under normal incidence, with the electric field polarized along the x-direction, and the antenna feed port was terminated by a 50 Ω matched load. A 416 mm × 416 mm metallic plate was first measured at the same location and used as a common 0 dB free-space normalization reference. A separate background response was obtained by covering the target region with pyramidal microwave absorbers. The complex S_11_(f) responses of the reference antenna and the proposed FP antenna were background-corrected and processed using time-domain gating around the target echo, whose nominal round-trip delay is approximately 16.7 ns for the 2.5 m range. Let $S_{11,ref}^{g,c}(f)$ and $S_{11,FP}^{g,c}(f)$ denote the gated and background-corrected complex responses, respectively. The measured finite-range backscattering reduction is calculated as follows:

$${\Delta R}_{meas}(f)=20{log}_{10}\left|\frac{S_{11,ref}^{g,c}(f)}{S_{11,FP}^{g,c}(f)}\right|$$

where the superscripts g and c denote time gating and background correction, respectively. Because both antenna measurements use the same metallic plate normalization, the plate term cancels in the final ratio. The reference antenna therefore provides the baseline for the reported reduction, whereas the metallic plate is used only for common free-space normalization.

The available 2.5 m range does not satisfy the conventional Fraunhofer criterion for the 416 mm aperture over the complete high-frequency scattering band, and no near-field-to-far-field transformation was applied. Therefore, the measured curve in Figure 18b is interpreted as a finite-range comparative backscattering reduction result, rather than as an absolute far-field RCS measurement. The full-wave far-field simulation gives a continuous 10 dB RCS reduction band of 3.05–6.47 GHz, whereas the measured finite-range backscattering reduction exceeds 10 dB from 3.15 to 6.32 GHz. The measured lower edge is shifted upward by about 0.10 GHz and the upper edge downward by about 0.15 GHz relative to simulation; thus, the measured band is slightly narrower but follows the same broadband suppression trend. The maximum measured reduction is approximately 24.2 dB near 5.2 GHz. Table 1 therefore uses the simulated far-field 10 dB RCS reduction band for this work, while the finite-range measurement provides comparative experimental validation.

Table 1 provides a contextual comparison of representative low-scattering FP antennas. Aperture efficiency is recalculated using the gain quantity and normalization frequency available from each source; however, ordinary gain and realized gain are not treated as interchangeable when the original source explicitly distinguishes them. Accordingly, the gain convention and aperture-normalization frequency are identified in the table notes where the source permits. Exact harmonization is not possible for every reference because the original publications do not always report the same gain convention or all quantities required for recalculation. The electrical aperture is listed because aperture efficiency is strongly size-dependent. The proposed antenna combines a compact 1.367λ_0_ × 1.367λ_0_ aperture with a measured gain-based aperture efficiency of 85.1%; this value therefore represents high aperture utilization of a compact aperture, rather than an aperture-independent superiority metric.

## 5. Conclusions

A high-aperture-efficiency FP antenna with broadband out-of-band RCS reduction has been developed using a cavity-oriented PRAFSS. Around 985 MHz, the PRAFSS provides the reflection magnitude and phase required for FP resonance, while absorbing only approximately 4.3% of the antenna-side incident power. The measured −10 dB impedance bandwidth is 965–1009 MHz (4.46%), the peak measured gain is approximately 13.0 dBi, and the measured gain enhancement is greater than 2.2 dB relative to the reference antenna. Using the exact 416 mm × 416 mm aperture, the measured gain-based aperture efficiency is approximately 85.1%. The designed scattering function is intentionally out of band: far-field simulations provide a continuous 10 dB monostatic RCS reduction band of 3.05–6.47 GHz, while finite-range measurements show a consistent comparative backscattering reduction band of 3.15–6.32 GHz. The results demonstrate that compact-aperture utilization and broadband out-of-band scattering suppression can be coordinated within a single PRAFSS-based FP architecture.

## Figures and Tables

**Table 1:** Performance comparison of representative low-scattering Fabry–Perot antennas.

Ref.	Mechanism	−10 dB Impedance BW (%)	Peak Gain Used for *η_ap_* (dBi)	Aperture (λ_0_ × λ_0_)	*η_ap_* (%)	Height (λ_0_)	10 dB Monostatic RCS Reduction Band
[24]	PRAS + absorber	11.1	12.3	2.55 × 2.55	20.8	0.67	N.R.
[19]	Absorbing surface + PRS + HIS	2.6	9.5	2.68 × 2.68	9.9	0.44	N.R.
[25]	PRAM + metamaterial ground	6.1	10.7	1.58 × 1.58	37.5	0.64	Dual bands (15% and 37% FBW)
[26]	Nonuniform PRS/phase cancellation	32.86	15.5	1.96 × 1.96	73.3	0.60	N.R.
[27]	Bandpass ATFSS	4.2	11.77	1.52 × 1.52	51.7	0.85	TE: 5–5.33, 9–13.4; TM: 8.4–13.5 GHz
[22]	FSRP + reflective metasurface	N.R.	21.8	4.8 × 4.8	52.3	N.R.	9.9–11.3 GHz ^S^
[23]	Single-layer PRS/phase cancellation	30.7	15.3	2.4 × 2.4	46.8	N.R.	N.R.
[28]	APRS + AMC	3.3	13.8	2.84 × 2.84	23.6	0.34	10.5–15.2, 22.5–28 GHz ^S^
This work	PRAFSS	4.46	13.0	1.367 × 1.367	85.1	0.61	3.05–6.47 GHz ^S^

Notes: This table is intended as a contextual comparison. Unless otherwise specified, tabulated performance values are measured results. Superscript S denotes simulated results; N.R. denotes that the corresponding quantity or a strict 10 dB RCS reduction band is not explicitly reported in the source. Gain convention/aperture-normalization frequency, where identifiable from the original source: ref. [24] gain/7.65 GHz; ref. [19] measured gain/10.7 GHz; ref. [25] measured gain/6.6 GHz; ref. [26] realized gain/9.2 GHz; ref. [27] measured gain/7.6 GHz; ref. [22] measured gain/12 GHz; ref. [23] measured gain/N.R.; ref. [28] realized gain/10.5 GHz; this work, measured gain/0.985 GHz. Aperture efficiencies are recalculated using Equation (2), with the gain quantity, physical aperture, and normalization frequency associated with each entry when these data are available. For ref. [28], the reported 81.2 mm aperture dimension and 13.8 dBi realized gain at 10.5 GHz yield an electrical aperture of 2.84λ_0_ × 2.84λ_0_ and an aperture efficiency of 23.6%. For this work, the finite-range measured comparative backscattering reduction band is 3.15–6.32 GHz; the far-field 10 dB RCS reduction band used in the table is the simulated 3.05–6.47 GHz result.

## Data Availability

The original contributions presented in this study are included in the article. Further inquiries can be directed to the corresponding author.
